# A diverse set of miRNAs responsive to begomovirus-associated betasatellite in *Nicotiana benthamiana*

**DOI:** 10.1186/1471-2229-14-60

**Published:** 2014-03-11

**Authors:** Bingguang Xiao, Xiuling Yang, Chu-Yu Ye, Yang Liu, Chenhai Yan, Yu Wang, Xiuping Lu, Yongping Li, Longjiang Fan

**Affiliations:** 1 Yunnan Academy of Tobacco Agricultural Sciences and China Tobacco Breeding Research Center at Yunnan, Yuxi 653100, China; 2 Department of Agronomy & James D. Watson Institute of Genome Sciences, Zhejiang University, Hangzhou 310058, China; 3 State Key Laboratory of Rice Biology, Institute of Biotechnology, Zhejiang University, Hangzhou 310058, China

**Keywords:** *Nicotiana benthamiana*, miRNA, phasiRNA, Begomovirus

## Abstract

**Background:**

Roles of microRNAs (miRNAs) and short interfering RNAs (siRNAs) in biotic stress responses, e.g., viral infection, have been demonstrated in plants by many studies. Tomato yellow leaf curl China virus (TYLCCNV) is a monopartite begomovirus that can systemically infect *Solanaceae* plants, and induces leaf curling, yellowing and enation symptoms when co-inoculated with a betasatellite (TYLCCNB). The released genome sequence of *Nicotiana benthamiana* provides an opportunity to identify miRNAs and siRNAs responsive to begomovirus-associated betasatellite in *N. benthamiana*.

**Results:**

miRNAs were identified in three small RNA libraries generated using RNA isolated from *N. benthamiana* plants systemically infected with TYLCCNV (Y10A) alone, co-infected with Y10A and its betasatellite TYLCCNB (Y10β) or a TYLCCNB mutant (Y10mβ) that contains a mutated *βC1*, the sole betasatellite-encoded protein. A total of 196 conserved miRNAs from 38 families and 197 novel miRNAs from 160 families were identified. Northern blot analysis confirmed that expression of species-specific miRNAs was much lower than that of conserved miRNAs. Several conserved and novel miRNAs were found to be responsive to co-infection of Y10A and Y10β but not to co-infection of Y10A and Y10mβ, suggesting that these miRNAs might play a role unique to interaction between Y10β and *N. benthamiana*. Additionally, we identified miRNAs that can trigger the production of phased secondary siRNAs (phasiRNAs).

**Conclusions:**

Identification of miRNAs with differential expression profiles in *N. benthamiana* co-infected with Y10A and Y10β and co-infected with Y10A and Y10mβ indicates that these miRNAs are betasatellite-responsive. Our result also suggested a potential role of miRNA-mediated production of phasiRNAs in interaction between begomovirus and *N. benthamiana*.

## Background

Endogenous small RNAs, which consist of microRNAs (miRNAs) and short interfering RNAs (siRNAs), are important regulators of organ development, stress responses and genomic stability in plants [1,2]. Roles of miRNAs and siRNAs in biotic and abiotic stress responses, e.g., viral infection, have been demonstrated in plants by many studies [2]. miRNAs downregulated or upregulated in plants infected with virus, such as miR156, miR158, miR159, miR160, miR164, miR168, miR171, miR172, miR319, miR398 and miR1885, might play a role in interactions between virus and plants [3-10]. Plant endogenous siRNA-mediated gene silencing was found to be involved in plant biotic stress, for example, bacteria-induced *nat-siRNAATGB2* regulates *R*-gene mediated plant immunity [11]. Rice stripe virus infection enhanced the accumulation of phased miRNAs from a particular precursor [12].

Plant miRNAs can trigger the production of phased secondary siRNAs (phasiRNAs) from either non-coding (e.g., *TAS*) or protein-coding genes (e.g. *NBS-LRR* genes). Trans-acting siRNAs (tasiRNAs) are distinctive siRNAs, which are generated from *TAS* transcripts in 21-nucleotide (nt) phases in relative to the miRNA cleavage site. They act in *trans* to regulate gene expression at the posttranscriptional level. Biogenesis of tasiRNAs is triggered by interaction of miRNA(s) at single or dual ends of the precursor *TAS* transcripts [13]. There are at least eight *TAS* loci that belong to four *TAS* families (*TAS1*–*TAS4*) in *Arabidopsis* and four *TAS3* loci have been identified in rice [13-15]. Recent studies demonstrated that plant genomes are rich in phased siRNA (phasiRNA)-producing loci, or *PHAS* genes [16-19], and may harbor hundreds of these loci in protein-coding genes [15,20-23]. For example, a batch of 21-nt and 24-nt phasiRNAs triggered by miR2118 and miR2275, respectively, were identified in the developing inflorescence of rice [18]. Biogenesis of phasiRNAs and their roles in posttranscriptional regulation have been well discussed in a recent review [24].

Geminiviruses are a group of single-stranded DNA viruses that cause devastating economic losses worldwide. Four genera (*Mastrevirus*, *Begomovirus*, *Curtovirus* and *Topocuvirus*) have been classified according to genome organizations, host ranges and transmission vectors, with *Begomovirus* being the most numerous and geographically wide-spread [25]. Begomoviruses have either monopartite or bipartite genomes. In the past decade, several studies have shown that many monopartite begomoviruses have acquired a betasatellite, formerly called DNAβ, and *βC1*, the sole betasatellite-encoded protein, plays essential roles in symptom induction, in suppression of transcriptional and posttranscriptional gene silencing, and also involves in suppression of selected jasmonic acid responses [25-29]. Tomato yellow leaf curl China virus (TYLCCNV) is a monopartite begomovirus associated with a betasatellite (TYLCCNB). TYLCCNV alone produced asymptomatic infection in *Nicotiana benthamiana* and tomato plants. TYLCCNB is essential for induction of leaf curl symptoms in these host plants when co-infected with TYLCCNV. TYLCCNB mutants unable to express the *βC1* protein abolished its ability to induce typical symptoms in plants when co-inoculated with TYLCCNV [27]. In our previous study, we identified TYLCCNV- and TYLCCNB-derived siRNAs by deep sequencing small RNAs from systemically infected *N. benthamiana* plant leaves and found that viral transcript might act as RNA dependent RNA polymerase (RDR) substrates resulting in secondary siRNA production [30]. However, plant miRNA profiles regulated by TYLCCNB remain largely unknown. Recent release of the draft genome sequence of *N. benthamiana *[31] provides an opportunity to identify miRNAs and siRNAs from the host plant and investigate expression changes of host miRNAs and siRNAs in response to viral infection. In this study, we identified *N. benthamiana* miRNAs based on the draft genome and our previously generated small RNA datasets using *N. benthamiana* plants infected with begomovirus, and found a number of miRNAs, including a few putative phasiRNA triggers, were responsive to co-infection of TYLCCNV and TYLCCNB but not to TYLCCNV and a mutated TYLCCNB in *N. benthamiana*.

## Results

### Identification of miRNAs in *N. benthamiana*

**Figure 1:**
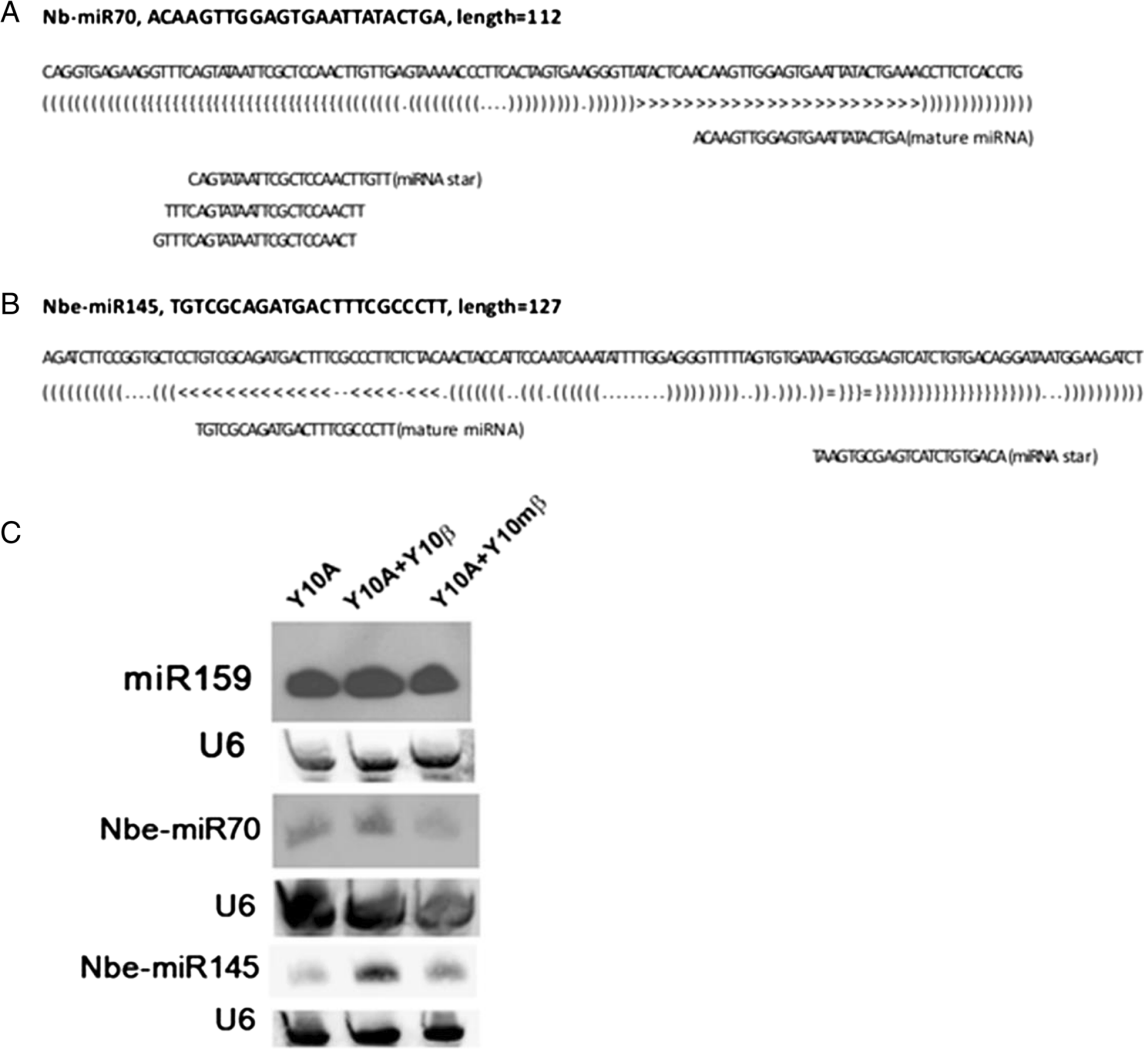
**Novel *Nicotiana benthamiana *miRNAs responsive to TYLCCNV/TYLCCNB infection. (A)** and **(B)** Precursors of two novel *N. benthamiana* miRNAs, Nbe-miR70 and Nbe-miR145. **(C)** Northern blot analysis of expression of Nbe-miR70 and Nbe-miR145 in *N. benthamiana* plants infected by TYLCCNV alone (Y10A or P1), together with TYLCCNB (Y10β or P2) or mutated TYLCCNB (Y10mβ or P3). Conserved miR159 was used as a miRNA control.

The *N. benthamiana* plant leaves systemically infected with TYLCCNV (Y10A) alone (P1), or co-infected with TYLCCNV and its betasatellite TYLCCNB (Y10β; P2) or a TYLCCNB mutant (Y10mβ; P3) were harvested for RNA extraction and small RNA sequencing [30]. In the 12.2 million small RNA reads or 7.3 million unique reads from *N. benthamiana* leaves, a total of 7.2 million reads or 5.6 million unique reads could be mapped to the *N. benthamiana* genome (Additional file 1: Table S1). Using the approach described in the Methods and based on BLAST search against the public miRNA database (miRBase, Release 19), we identified a total of 196 conserved miRNAs from 38 families in the *N. benthamiana* genome (Additional file 2: Table S2). Meanwhile, a total of 197 novel miRNAs from 160 families were also identified (Additional file 3: Table S3). A further search (BLASTn with < 1e^-3^) of these novel mature miRNA sequences against the small RNA populations from *N. tabacum* recently generated by us [32] showed that 42 novel *N. benthamiana* miRNAs is conserved in *N. tabacum*. Target prediction indicated 147 (91.9%) novel miRNAs had at least one potential target (Additional file 3: Table S3). Expression of two novel miRNAs, Nbe-miR70 and Nbe-miR145, together with the conserved miR159 could be detected by Northern blot, although the expression levels of the two novel miRNAs were much lower than that of miR159 (Figure 1), consistent with the well known phenomenon for lowly expressed non-conserved miRNAs.

### Identification of putative phasiRNA triggers

**Figure 2:**
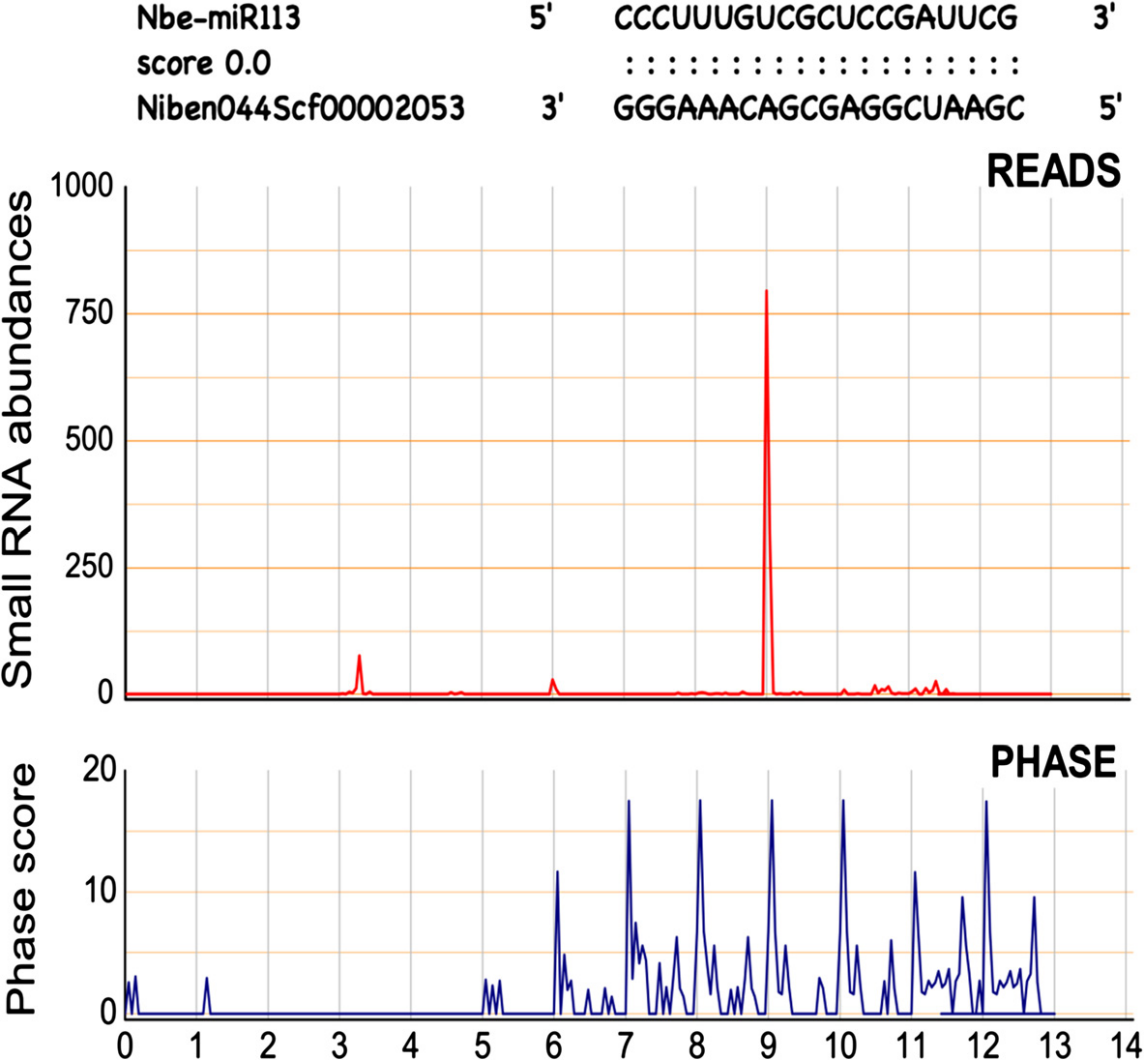
**Nbe-miR113 triggered phasiRNA production in an *NBS-LRR *gene (Niben044Scf0002438) in *N. benthamiana*. (A)** The alignment between miRNA and its target. Two dots indicate matches. **(B)** The small RNA abundances and phasing score distributions across Niben044Scf0002438.

Based on the draft genome of *N. benthamiana* and the algorithm described by Howell et al. [17] (see Methods for the details), we found that over thousands of loci were able to generate 21-nt and 24-nt phasiRNAs (phase score > 1.4). Of these phasiRNA loci, miRNA binding site(s) could be predicted in at least 157 of 21-nt and 296 of 24-nt phasiRNA loci (within the region with phased small RNA reads and their two flanking 200-nt regions), suggesting that these miRNAs are putative phasiRNA triggers in *N. benthamiana* (Additional file 4: Table S4). For these potential phasiRNA triggers or phase registers, their putative cleavage sites (between positions 10 and 11 in the predicted miRNA target sites) were all located at a phase position. A total of 62 conserved miRNAs from 24 families which could bind to *PHAS* loci were found, e.g., miR156 and miR482 (Table 1). More than 300 *PHAS* loci seem to be triggered by *N. benthamiana* or *Nicotiana*-specific miRNAs, such as Nbe-miR2 (Additional file 4: Table S4). One example was shown in Figure 2, in which Nbe-miR113, a novel *N. benthamiana* miRNA, was predicted to trigger production of 21-nt phased small RNAs in a *N. benthamiana* gene. In addition to non-coding transcript, such as *TAS* genes, many protein-coding genes were also identified as miRNA-triggered *PHAS* loci in *N. benthamiana* (Additional file 4: Table S4), including some loci targeted by conserved miRNAs, such as miR156 and miR482 (Table 1), which have been shown to generate phasiRNAs from protein-coding genes in several recent studies [15,20-23,33,34]. When an extremely strict phase score (> 20) was applied, 1 and 13 loci generating 21-nt and 24-nt phasiRNAs, respectively, could still be identified.

**Table 1:** **Selected miRNAs (including some as *PHAS *** **trigger) with differentially expression changes in response to co-infection of TYLCCNV and TYLCCNB in *N. benthamiana***

**miRNA family**	Expression change (fold)*	**Predicted target**	**References for virus response**	**As *PHAS *** **trigger#**	**References for *PHAS***
**Conserved**					
miR160	↑7.6	Auxin response factor	[3,8,9]	ND	
miR164	↑2.2	NAC domain protein	[3,7,9]	ND	
miR169	↓2.0	CCAAT-binding transcription factor (CBF)	[3,8]	ND	
miR319	↓2.0	TCP/MYB	[8]	Yes	
miR391	↓3.5	Putative *TAS*	[8]	Yes	
miR396	↑2.4	LRR receptor-like serine/threonine-protein kinase, RLP/Pentatricopeptide repeat protein	[8]	Yes	
miR397	↑3.5	Laccase	[8]	Yes	
miR398	↑18.4	CSD; Zinc finger, LIM-type/Rhodanese-like	[8,10]	Yes	
miR482 /miR2118	↑11.0	NBS-LRR	[21]	Yes	[15,18,21]
miR827	↓3.1	AP2 domain-containing transcription factor/RCC1-like protein		Yes	
miR4376	↓3.7	ACA		Yes	[22]
miR6020	↓2.0	Pentatricopeptide repeat/Glycosyl transferase	[20]	Yes	[20]
**Novel**					
Nbe-miR2	↓5.9	RCC domain-containing protein		ND	
Nbe-miR3	↓6.7	Not known		ND	
Nbe-miR4	↓3.8	Not known		ND	
Nbe-miR6	↑2.8	GH3 family protein		ND	
Nbe-miR8	↑2.3	Heat shock protein binding protein		ND	
Nbe-miR64	↑2.1	C2 membrane targeting protein		Yes	
Nbe-miR70	↑2.1	Basic helix-loop-helix dimerisation region bHLH		ND	
Nbe-miR84	↑3.4	Cyclic nucleotide gated channel		ND	
Nbe-miR99	↑3.5	F-box family protein		Yes	
Nbe-miR107	↑9.1	DNA repair protein radA		ND	
Nbe-miR124	↑2.9	Lactoylglutathione lyase		ND	
Nbe-miR129	↑2.1	Harpin-induced protein-like		ND	
Nbe-miR134	↑2.8	CHP-rich zinc finger protein/F-box family protein		Yes	
Nbe-miR142	↓2.0	Receptor like kinase, RLK		ND	

References for virus response and *PHAS* by previous studies were listed. A detailed list was shown as Additional file 5: Table S5.

*Fold of P2/P1; #ND: not determined.

### Expression profiles of miRNAs responsive to co-infection of TYLCCNV/ TYLCCNB

**Figure 3:**
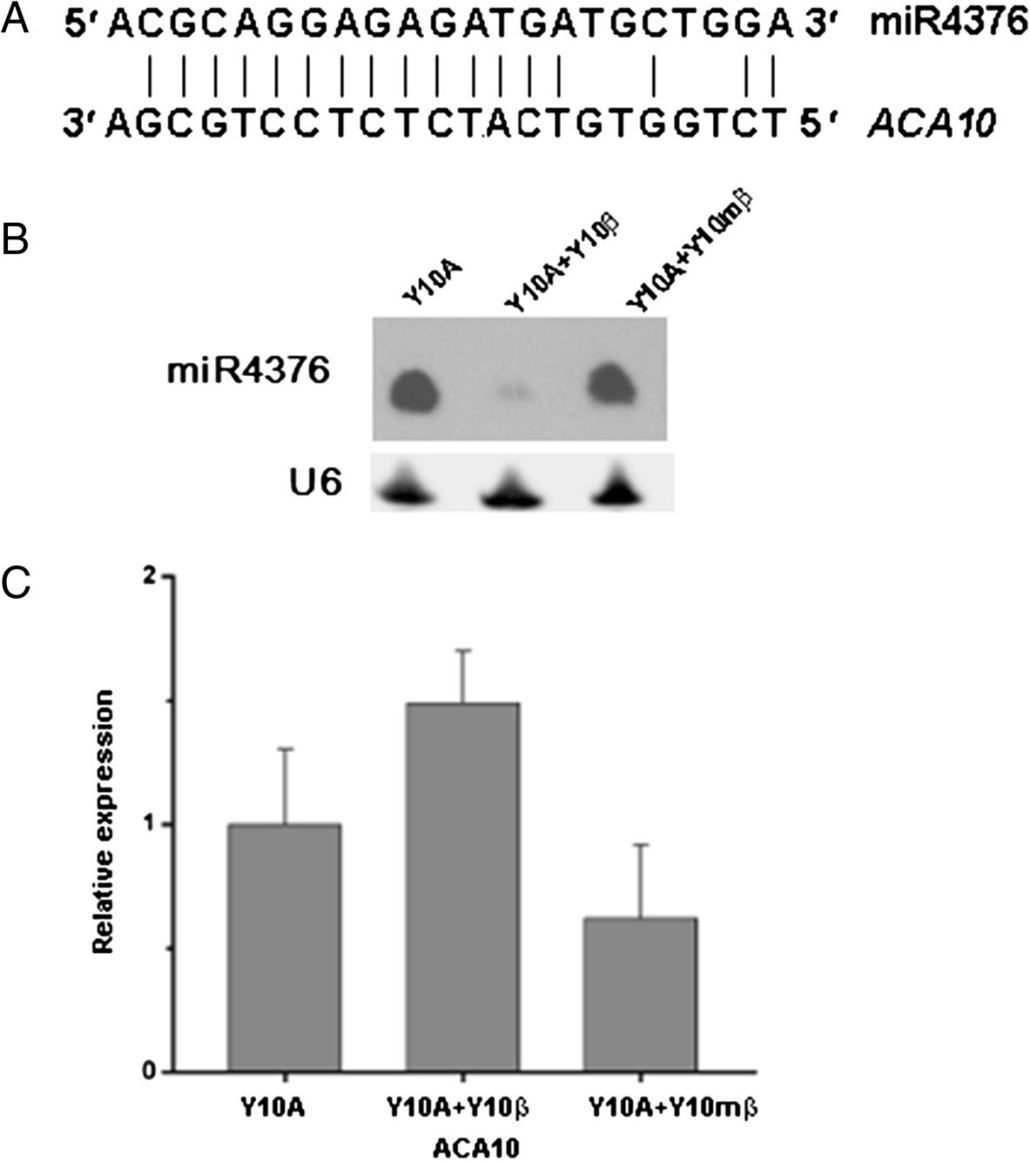
**Expression of miR4376 and its target (*ACA10*) mRNA in three different cDNA libraries of *N. benthamiana*. (A)** Alignment of miR4376 with its target *ACA10*. **(B)** Northern blotting of miR4376 in *N. benthamiana* plants infected by TYLCCNV alone (Y10A or P1), together with TYLCCNB (Y10β or P2) or mutated TYLCCNB (Y10mβ or P3). **(C)** Expression of *ACA10* in various treatments measured by Real-time qPCR. Error bars represent standard deviation determined with three biological replicates.

Previous reports showed that some miRNAs were differentially expressed after virus infection [8,10]. In this study, our focus was on the miRNAs responsive to betasatellite (TYLCCNB) associated with TYLCCNV, i.e. those with a similar expression level in plants infected with TYLCCNV (Y10A) and co-infected with TYLCCNV and a mutated TYLCCNB (i.e. Y10A + Y10mβ) but with an up- or down-regulated expression level in plants co-infected with TYLCCNV and a functional TYLCCNB (i.e. Y10A + Y10β). With the criteria of 1.7-fold changes in Y10A + Y10β versus both Y10A and Y10A + Y10mβ and RPM (reads per million) ≥ 10 in at least one of the three samples, we found that among the conserved miRNAs, miR160, miR164, miR397 and miR398 were up-regulated whereas miR169, miR391 and miR827 were down-regulated in responsive to TYLCCNB, and that among the novel miRNAs, Nbe-miR8, Nbe-miR70, Nbe-miR107, Nbe-miR124 and Nbe-miR134 were up-regulated whereas Nbe-miR2, Nbe-miR3 and Nbe-miR142 were down-regulated in responsive to TYLCCNB (Table 1; Additional file 5: Table S5). Up-regulation of Nbe-miR70 was confirmed by northern blot (Figure 1). In addition, down-regulation of miR4376 observed in plants co-infected with TYLCCNV and a functional TYLCCNB, i.e. Y10A + Y10β, was also confirmed by the northern blot results; consequently, an up-regulation of its target gene *ACA10* (*autoinhibited Ca*^*2+*^*-ATPase 10*) was detected by RT-qPCR (Figure 3; Additional file 5: Table S5).

The majority of miRNAs had a similar expression level in *N. benthamiana* plants systemically infected with TYLCCNV alone (P1) and co-infected with TYLCCNV and a mutant betasatellite TYLCCNB (P3) (Additional file 5: Table S5), confirming an un-functional *βC1* in the mutated TYLCCNB. This result also suggests that the aforementioned miRNAs that were responsive to betasatellite most likely indeed have a role in interaction between begomovirus and *N. benthamiana*. Interestingly, some of the differentially expressed miRNAs, such as miR391, miR397 and miR398, were putative triggers of phasiRNA production (Table 1).

## Discussion

The involvement of miRNAs in diverse biotic responses (viral, bacterial and fungal) has been demonstrated by recent studies [2]. Our study confirmed that the expression of host miRNAs (such as miR391, miR397 and miR398) is affected in response to betasatellite. There are at least two different kinds of miRNAs: one is suppressed by the viral suppressors of RNA silencing (VSRs) while another is involved in anti-pathogen (virus) by regulating plant innate immune receptors or hormone signaling pathway. Viral encoded proteins interfere with host RNAi pathways and thus distort the normal cellular activities [8]. They can bind directly to host miRNAs and therefore making non-function of RNA-induced silencing complex (RISC). miRNAs are key regulatory molecules in diverse biological processes in plants, for example, miR164, miR159/319 for leaf development [1]. Apparently, the typical leaf curl symptoms in *N. benthamiana* induced by co-infection of TYLCCNV and its betasatellite TYLCCNB could be caused by deregulation of those differentially accumulated miRNAs in responsive to betasatellite (Table 1). The differentially down-regulated miR4376 in *N. benthamiana* plants co-infected with TYLCCNV and TYLCCNB was predicted to target an *ACA* gene and could also be involved in leaf development, because a recent study showed that miR4376 targets *ACA10* gene and plays a critical role in tomato reproductive growth [22]. In addition, the miRNAs, such as miR160, that were up-regulated in plants co-infected with TYLCCNV and a mutated TYLCCNB but the up-regulation level was much smaller than that in plants co-infected with TYLCCNV and a functional TYLCCNB could also have a role in response to betasatellite.

Recent studies demonstrated a role of miRNA-mediated phasiRNA pathway in diverse biological processes, including pathogen resistance [15,20-23,34]. For example, miR482/2118-mediated cleavage of disease resistance *NBS-LRR* genes not only plays an important role in non-race specific disease resistance but triggers production of phased small RNAs that are able to regulate the expression of their targets in *trans *[15,21]. Upon pathogen infection, the expression level of miR482 was down-regulated and consequently its targets, *NBS-LRR* genes, were up-regulated [21,34]. miR482/2118 belongs to a specific type of miRNA that generated from pre-miRNAs containing asymmetric bulges in the miRNA/miRNA* duplex and has been demonstrated to be the trigger for production of phased 21-nt secondary small RNAs from their target transcripts through the RDR6/DCL4 pathway [34]. Therefore, miR482/2118-mediated cleavage of target disease resistance genes is expected to cause both decay of their target mRNAs and production of phased secondary small RNAs from the target genes, such as shown in tomato, cotton and *Medicago truncatula *[15,21,34]. Furthermore, at least one of the secondary small RNAs generated from a miR482 targeted *NBS-LRR* gene has been shown to target mRNA encoding another defense-related protein [21], indicating that the secondary small RNAs generated from protein-coding genes can behave like *TAS* loci in *Arabidopsis *[34]. In this study, the expression level of miR482 was up-regulated in *N. benthamiana* plants co-infected with TYLCCNV and TYLCCNB compared to plants infected with TYLCCNV alone, which is in contrast with previous observation. Meanwhile, this up-regulation of miR482 was also observed in plants co-infected with TYLCCNV and a mutated TYLCCNB (Additional file 5: Table S5). Therefore, more investigation is required to understand the function of miR482 in response to betasatellite. In addition to miR482, several other betasatellite-responsive miRNAs, such as miR397 and miR398, were potential triggers of phasiRNAs (Additional file 4: Table S4), suggesting that both these miRNAs and the phasiRNAs generated from targets of these miRNAs could play a role in interaction between begomovirus and *N. benthamiana*.

## Conclusions

A total of 196 conserved miRNAs and 197 novel miRNAs were found in the genome of *N. benthamiana*. Among them, several showed a differential expression pattern in *N. benthamiana* plants co-infected with TYLCCNV and TYLCCNB and co-infected with TYLCCNV and a mutated TYLCCNB, suggesting a role of these miRNAs in interaction between the betasatellite associated with begomovirus and *N. benthamiana* plants. In addition, our study showed that several of these differentially expressed miRNAs were potential triggers for production of phasiRNAs.

## Methods

### Small RNA and genomic data

Three sets of small RNA populations (accession number: GSE26368) from *N. benthamiana* were generated in our previous study using high-throughput sequencing technology [30]. In short, *N. benthamiana* plant leaves systemically infected with Tomato yellow leaf curl China virus (TYLCCNV) alone (P1), co-infected with TYLCCNV and its associated betasatellite (TYLCCNB) (P2) or mutated TYLCCNB (P3) were sampled and used in sRNA cDNA library construction. The sRNA libraries were sequenced using high-throughput sequencing technology. Draft genomic sequences of *N. benthamiana* were downloaded from http://solgenomics.net (Niben.genome.v0.4.4) [31].

### Annotation of miRNAs and loci generating phasiRNAs

All small RNA data were processed by a suite of perl scripts. Clean reads were mapped to the *N. benthamiana* genome sequences. Reads mapped to house-keeping noncoding RNAs (e.g., rRNA, tRNA, snRNA, snoRNA) were excluded for further analysis. Our previous bioinformatic pipeline designed following the criteria previously described by Meyers et al. [35] was applied in miRNA annotation [36]. Prediction of stem-loop structure was performed by the Vienna RNA package [37]. Candidates (pre-miRNAs) with an ideal hairpin structure containing the pair of small RNAs that are able to form a miRNA::miRNA^*^ duplex with less than 4 mismatches and 2-nt of 3′ overhangs were selected. Targets of miRNAs were predicted by searching against the coding regions of the *N. benthamiana* genome using psRNATarget (http://plantgrn.noble.org/psRNATarget/) [38] with the default settings (maximum expectation: 3.0; length for complementary scoring: 20 bp; target accessibility - allowed maximum energy to unpair the target site: 25.0; flanking length around target site for target accessibility analysis: 17 bp in upstream and 13 bp in downstream; range of central mismatch leading to translational inhibition: 9–11 nt). *PHAS* loci were identified using the algorithm

$$P=\mathrm{In}\left[{\left(1+\sum_{i=1}^{8}k_{i}\right)}^{n-2}\right],P>0,$$

(where *n* = number of phase cycle positions occupied by at least one small RNA read within an eight-cycle window, and *k* = the total number of reads for all small RNAs with consolidated start coordinates in a given phase within an eight-cycle window) described by Howell et al. [17] and our previous studies [19,32]. miRNAs that bind to *PHAS* loci within the regions generating phased small RNA reads or their flanking 200-nt regions were considered as putative phasiRNA triggers. In these *PHAS* loci, the putative miRNA cleavage sites (between positions 10 and 11 in the predicted miRNA target sites) were always found at a position corresponding to a phase register.

### Northern blot analysis of miRNAs

Total RNA was extracted from systemically-infected *N. benthamiana* leaves using Trizol Reagents following the manufacturer’s instructions (Invitrogen, Carlsbad, CA). A total of 15–30 μg low molecular weight (LMW) RNA, isolated by Polyethyleneglycol (molecular weight 8000, PEG8000) and NaCl [39], was separated on a denaturing 15% polyacrylamide denaturing gels and transferred to Hybond N^+^ membrane. After being cross-linked by UV radiation, the Hybond membrane was hybridized with biotin-labeled DNA probes complementary to candidate miRNA sequences at 42°C overnight. Post hybridization washes were performed twice using 1X SSC and 0.5% sodium dodecyl sulfate (SDS) at 42°C for 15 min. Hybridization signals were detected with stabilized streptavidin-horseradish peroxidase conjugate supplemented in Chemiluminescent Nucleic Acid Detection Module (Thermo, Pierce) according to the manufacturer’s instructions. To confirm equal loading of the gels, U6 was selected as internal control and blots were also hybridized with a DNA probe complementary to U6.

### Real-time quantification of miRNA target

One μg of DNase I-treated total RNA was reverse transcribed using AMV Reverse Transcriptase (Takara, Dalian, China) following the manufacturer’s instructions. Quantitative real-time PCR was performed using SYBR Green PCR supermix (Roche, Mannheim, Germany) in a Lightcycler® Real-time PCR system (Roche) as described [30]. Three independent biological replicates were conducted and the relative expressions of miRNA target gene were normalized using the CT values obtained for the internal control *GADPH*. Analysis was carried out using Lightcycler® 480 software supplemented by the manufacturer (Roche).

## Abbreviations

miRNA: microRNA; siRNA: Short interfering RNA; phasiRNA: Phased, secondary siRNA; NBS: Nucleotide binding site; tasiRNA: Trans-acting siRNA.

## Competing interests

The authors declare that they have no competing interests.

## Authors’ contributions

BX and LF conceived the study. XY performed experimental validation of miRNAs and RT-qPCR analysis. C-Y Y, YL, CY and YW identified miRNAs and phasiRNAs and performed expression analysis. LF, BX, XL, and YL discussed the data. LF, XY and C-Y Y wrote the manuscript. All authors read and approved the final manuscript.

## Supplementary Material

Additional file 1: Table S1 Statistics of small RNAs which can be mapped to the *N. benthamiana* genome. The *N. benthamiana* plant leaves systemically infected with TYLCCNV alone (P1), or together with betasatellite TYLCCNB (P) or with mutant TYLCCNB (P3) were harvested for RNA extraction and small RNA sequencing. Click here for file

Additional file 2: Table S2 Detailed information of conserved miRNAs identified in *N. benthamiana* by this study. Click here for file

Additional file 3: Table S3 Detailed information of novel miRNAs identified in *N. benthamiana* by this study. Click here for file

Additional file 4: Table S4 Detailed information of miRNAs as putative *PHAS* triggers in *N. benthamiana*. Click here for file

Additional file 5: Table S5 List of miRNAs with significant expression changes in *N. benthamiana* after TYLCCNV affection. Click here for file
