# Supplementary material for: A diverse set of miRNAs responsive to begomovirus-associated betasatellite in Nicotiana benthamiana
Source: BMC Plant Biol. 2014 Mar 11;14:60. doi: 10.1186/1471-2229-14-60 (PMC4008317; doi:10.1186/1471-2229-14-60)
Supplement: Additional file 1: Table S1 — Statistics of small RNAs which can be mapped to the N. benthamiana genome. The N. benthamiana plant leaves systemically infected with TYLCCNV alone (P1), or together with betasatellite TYLCCNB (P) or with mutant TYLCCNB (P3) were harvested for RNA extraction and small RNA sequencing. [file 1471-2229-14-60-S1.docx]

**Additoal file 1: Table S1** Statistics of small RNAs which can be mapped to the *N. benthamiana* genome. The *N. benthamiana* plant leaves systemically infected with TYLCCNV alone (P1), or together with betasatellite TYLCCNB (P) or with mutant TYLCCNB (P3) were harvested for RNA extraction and small RNA sequencing.

| Item | Small RNA population | | |  |  | Perfect map to the genome | | |  |
| --- | --- | --- | --- | --- | --- | --- | --- | --- | --- |
|  | P1 | P2 | P3 | Total |  | P1 | P2 | P3 | Total |
| Total reads | 3,621,688 | 4,291,983 | 4,346,499 | 12,260,170 |  | 2,175,906 | 2,469,439 | 2,643,668 | 7,289,013 |
| Unique reads | 2,243,738 | 2,478,346 | 2,595,034 | 7,317,118 |  | 1,696,940 | 1,891,187 | 2,043,466 | 5,631,593 |
